# Supplementary material for: Patterns of Intron Gain and Loss in Fungi
Source: PLoS Biol. 2004 Nov 30;2(12):e422. doi: 10.1371/journal.pbio.0020422 (PMC532390; doi:10.1371/journal.pbio.0020422)
Supplement: Table S1 — Also available at http://genes.mit.edu/NielsenEtAl/. (4.3 MB ZIP). [file pbio.0020422.st001.zip › NielsenEtAl/html/467.html]

AN0667.1.NCU07165.1.MG10677.1.FG04481.1


```
 CLUSTAL W (1.82) Multiple Sequence Alignments - Introns Inserted


Sequence 1: MG10677.1	447 aa
Sequence 2: FG04481.1	448 aa
Sequence 3: NCU07165.1	459 aa
Sequence 4: AN0667.1	461 aa
Alignment Length: 473 aa
Number Identitical Residues: 223 aa
Alignment Score (without introns) 10897


MG10677.1 	-MQVPLFRLQCG~VNSYDWGK~KGSESAAAKFAAATPAD-DFSIQDDTPYAE~LWMGTHP
NCU07165.1	MLPVPLIQLQCG~VNSYEWGK~KGNSSRAAQFAAASLDDNEFKIDEEKPYAE0LWMGTHP
FG04481.1 	-MQVPLFRLQCG~VNSYAWGK~KGDDSAAARFAAATPSD-ELKIESDTPYAE0LWMGTHP
AN0667.1  	-MQVPLLRLQCG1VNSYDWGR0VGPESAAAKYAAATAPS-DFTIEADKPYAE0LWMGTHP
          	 : ***::**** **** **:  * .* **::***:  . ::.*: :.**** *******

MG10677.1 	SNPSKDVDTGRTLLDLVEDNQMFLSSSVISKYGSKLPFLFKVLSINKALSIQAHPNKKLA
NCU07165.1	SNPSKDKHTGRTLLELIQDNQALLSPAIAERYQNKLPFLFKVLSIQKALSIQAHPNKKLA
FG04481.1 	SNPSRDLNTGRTLLELCEDNQALLSQTISSHYGNKLPFLFKVLSIAKALSIQAHPNKKLA
AN0667.1  	SLPSKDVETQRTLLDMVQDNLALMSPEVSERYGGKLPFLFKVLSIRKALSIQAHPNKKLA
          	* **:* .* ****:: :**  ::*  : .:* .*********** **************

MG10677.1 	EKLHERDPKNYPD~DNHKPEMAIAITPFEGLCGFRPLTEIAHFLDNVPALRSLVGEDKAK
NCU07165.1	EKLHARDPKNYPD~DNHKPEMAIAISDFEGLCGFRPLKEIAHFLDNVPALRQLVGEDKAK
FG04481.1 	EQLHARDPKNYPD~DNHKPEMAIAITPFEGLCGFRPLGEIAHFLDTVPALRALVGDEVAK
AN0667.1  	EALHARDPRNYPD1DNHKPEMTIAITPFEGLCGFRPLAEIVHFLKAVAPLRYLIGVQTAT
          	* ** ***:**** *******:***: ********** **.***. *..** *:* : *.

MG10677.1 	QFVEVAK----DEKADVAT-KKKALREAFAAVMDASESALAEATKQLVSAAESEGADFA-
NCU07165.1	AFVETVKRNDGNESDEATAENKKVLQSAFGALMASSEADMTAAADKLVQSAKSAGADFT-
FG04481.1 	EFAQVAK----DDEEGVTDEKKKLLKKAFGALMSSLAEDIAEQMPKLAEQAEKEGADFA-
AN0667.1  	DFENAVRGFENTEDPEQTKKNKVALRTLFTSLMQSASENIEQAARELVAAAQSSPETFAS
          	 * :..:  .. :.   : .:*  *:  * ::* :    :     :*.  *:.    *::

MG10677.1 	---AKGVSSTSGATLAELVNRLHGQFGADYGLFVLFYCNFVTLEPGEALFLQADDIHAYV
NCU07165.1	---GTAVPSPGGAKLSELVGRLHGQFGADYGLFVLFFLNFVEMKAGEAIYLRADDIHAYI
FG04481.1 	---GGGVVATSGEKLAELVKRTHGEFGDDIGCFVLFFLNFVTLEPGEALFLVADDIHAYI
AN0667.1  	LVNAPDTNPTNAAELASIIIRLNEQFPNDIGLFVFFFLNFVRLEPGEAMFLKADDIHAYI
          	  ..  . ....  *:.:: * : :*  * * **:*: *** ::.***::* *******:

MG10677.1 	SGDIMECMAASDNVVRAGFTPKFKDVPTLVDMLTYDSAPIDEQKMQPTEYPY--------
NCU07165.1	SGDIIECMASSDNVVRAGFTPKFKDVDNLVNMLTYDFAPIEQQKMEPTDYTQDVKNKREA
FG04481.1 	SGDIMECMAASDNVVRAGFTPKFKDVSTLVDMLTYNFAPIEEQKMTPTDYPY--------
AN0667.1  	SGDIIECMASSDNVVRAGFTPKFKDVDTLTEMLTYSYAPIDEQKLQPTDYPY--------
          	****:****:**************** .*.:****. ***::**: **:*.         

MG10677.1 	-VKLNMAAFKSGSEVLLYDPPIEEFSVLRGVLKANEAKATFDPVEGPSIVICTKGSGKIS
NCU07165.1	GATLNQTAIDSQSEIVEYNPPIEEFSVVRSLLKSQGSKVTFKPIDGPSIIICTQGQGKIS
FG04481.1 	-ATLNRAGYSSGSAVVLYDPPIEEFSVVRSVLRGDGSKATFDPLEGPSVVICTGGRGKIS
AN0667.1  	-TVLNAAAYSSASDSLLYDPPIEEFSVVKTSLRRTGAKATFDPLTGPSILICTGGTGKIS
          	 . ** :. .* *  : *:********::  *:   :*.**.*: ***::*** * ****

MG10677.1 	VGPTVKDIKEGWVYFVGSTAQCVLEADSL--GDGEEFHFFKAFCEVE---DNSNGA-
NCU07165.1	VGPKVQEIKKGHVFFVGATAECVLESED------DKFETFKAFCVLD---EQSNGN-
FG04481.1 	VGPTSAEMKEGYVYFVGATAELVLESEG---GEDDEFTTFKAFCEID---DSEKQKL
AN0667.1  	VGHKTEEVKEGYVFFVGANAECIIENTGTGSDEENVFTTFKAFCDLTGKEDMANGH-
          	** .  ::*:* *:***:.*: ::*  . .:.. : *  ***** : ...:  :
```
